# Supplementary material for: The Limitations of Model-Based Experimental Design and Parameter Estimation in Sloppy Systems
Source: PLoS Comput Biol. 2016 Dec 6;12(12):e1005227. doi: 10.1371/journal.pcbi.1005227 (PMC5140062; doi:10.1371/journal.pcbi.1005227)
Supplement: S1 Supporting Information — (ZIP) [file pcbi.1005227.s001.zip › MAModelDef.pdf]

I. MASS ACTION MODEL DEFINITION

$$\frac{d}{dt}[\mathrm{bNGFR}] = k_{1f}\ [\mathrm{NGF}]\ [\mathrm{fNGFR}] - k_{1b}\ [\mathrm{bNGFR}] - k_{4f}\ [\mathrm{bNGFR}]\ [\mathrm{SosI}] + k_{4b}\ [\mathrm{C_{bNGFR-Sos}}] + k_{5f}\ [\mathrm{C_{bNGFR-Sos}}] - k_{40f}\ [\mathrm{bNGFR}]\ [\mathrm{C3GI}] + k_{40b}\ [\mathrm{C_{bNGFR-C3G}}] + k_{41f}\ [\mathrm{C_{bNGFR-C3G}}] \tag{1}$$

$$\frac{d}{dt}[\mathrm{NGF}] = -k_{1f}\ [\mathrm{NGF}]\ [\mathrm{fNGFR}] + k_{1b}\ [\mathrm{bNGFR}] \tag{2}$$

$$\frac{d}{dt}[\mathrm{C_{Ras-PI3K}}] = k_{36f}\ [\mathrm{RasA}]\ [\mathrm{PI3KI}] - k_{36b}\ [\mathrm{C_{Ras-PI3K}}] - k_{37f}\ [\mathrm{C_{Ras-PI3K}}] \tag{3}$$

$$\frac{d}{dt}[\mathrm{ErkI}] = -k_{30f}\ [\mathrm{MekA}]\ [\mathrm{ErkI}] + k_{30b}\ [\mathrm{C_{Mek-Erk}}] + k_{33f}\ [\mathrm{C_{PP2A-Erk}}] \tag{4}$$

$$\frac{d}{dt}[\mathrm{MekA}] = k_{25f}\ [\mathrm{C_{Raf1-Mek}}] + k_{27f}\ [\mathrm{C_{BRaf-Mek}}] - k_{28f}\ [\mathrm{PP2AA}]\ [\mathrm{MekA}] + k_{28b}[\mathrm{C_{PP2A-Mek}}] - k_{30f}\ [\mathrm{MekA}]\ [\mathrm{ErkI}] + k_{30b}\ [\mathrm{C_{Mek-Erk}}] + k_{31f}\ [\mathrm{C_{Mek-Erk}}] \tag{5}$$

$$\frac{d}{dt}[\mathrm{C_{bNGFR-Sos}}] = k_{4f}\ [\mathrm{bNGFR}]\ [\mathrm{SosI}] - k_{4b}\ [\mathrm{C_{bNGFR-Sos}}] - k_{5f}\ [\mathrm{C_{bNGFR-Sos}}] \tag{6}$$

$$\frac{d}{dt}[\mathrm{C_{Raf1PPtase-Raf1}}] = k_{16f}\ [\mathrm{Raf1PPtase}]\ [\mathrm{Raf1A}] - k_{16b}\ [\mathrm{C_{Raf1PPtase-Raf1}}] - k_{17f}\ [\mathrm{C_{Raf1PPtase-Raf1}}] \tag{7}$$

$$\frac{d}{dt}[\mathrm{C_{BRaf-Mek}}] = k_{26f}\ [\mathrm{BRafA}]\ [\mathrm{MekI}] - k_{26b}\ [\mathrm{C_{BRaf-Mek}}] - k_{27f}\ [\mathrm{C_{BRaf-Mek}}] \tag{8}$$

$$\frac{d}{dt}[\mathrm{C_{bNGFR-C3G}}] = k_{40f}\ [\mathrm{bNGFR}]\ [\mathrm{C3GI}] - k_{40b}\ [\mathrm{C_{bNGFR-C3G}}] - k_{41f}\ [\mathrm{C_{bNGFR-C3G}}] \tag{9}$$

$$\frac{d}{dt}[\mathrm{C_{P90/RSK-Sos}}] = k_{6f}\ [\mathrm{P90/RSKA}]\ [\mathrm{SosA}] - k_{6b}\ [\mathrm{C_{P90/RSK-Sos}}] - k_{7f}\ [\mathrm{C_{P90/RSK-Sos}}] \tag{10}$$

$$\frac{d}{dt}[\mathrm{P90/RSKI}] = -k_{8f}\ [\mathrm{ErkA}]\ [\mathrm{P90/RSKI}] + k_{8b}\ [\mathrm{C_{Erk-P90/RSK}}] \tag{11}$$

$$\frac{d}{dt}[\mathrm{EGF}] = -k_{0f}\ [\mathrm{EGF}]\ [\mathrm{fEGFR}] + k_{0b}\ [\mathrm{bEGFR}] \tag{12}$$

$$\frac{d}{dt}[\mathrm{C_{Raf1PPtase-BRaf}}] = k_{22f}\ [\mathrm{Raf1PPtase}]\ [\mathrm{BRafA}] - k_{22b}\ [\mathrm{C_{Raf1PPtase-BRaf}}] - k_{23f}\ [\mathrm{C_{Raf1PPtase-BRaf}}] \tag{13}$$

$$\frac{d}{dt}[\mathrm{PP2AA}] = -k_{28f}\ [\mathrm{PP2AA}]\ [\mathrm{MekA}] + k_{28b}\ [\mathrm{C_{PP2A-Mek}}] + k_{29f}\ [\mathrm{C_{PP2A-Mek}}] - k_{32f}\ [\mathrm{PP2AA}]\ [\mathrm{ErkA}] + k_{32b}\ [\mathrm{C_{PP2A-Erk}}] + k_{33f}\ [\mathrm{C_{PP2A-Erk}}] \tag{14}$$

$$\frac{d}{dt}[\mathrm{Akt/PKBI}] = -k_{38f}\ [\mathrm{PI3KA}]\ [\mathrm{Akt/PKBI}] + k_{38b}\ [\mathrm{C_{PI3K-Akt}}] \tag{15}$$

$$\frac{d}{dt}[\mathrm{Akt/PKBA}] = -k_{18f}\ [\mathrm{Akt/PKBA}]\ [\mathrm{Raf1A}] + k_{18b}\ [\mathrm{C_{Akt-Raf1}}] + k_{19f}\ [\mathrm{C_{Akt-Raf1}}] + k_{39f}\ [\mathrm{C_{PI3K-Akt}}] \tag{16}$$

$$\frac{d}{dt}[\mathrm{C_{RasGap-Ras}}] = k_{12f}\ [\mathrm{RasGapA}]\ [\mathrm{RasA}] - k_{12b}\ [\mathrm{C_{RasGap-Ras}}] - k_{13f}\ [\mathrm{C_{RasGap-Ras}}] \tag{17}$$

$$\frac{d}{dt}[\mathrm{SosA}] = k_{3f}\ [\mathrm{C_{bEGFR-Sos}}] + k_{5f}\ [\mathrm{C_{bNGFR-Sos}}] - k_{6f}\ [\mathrm{P90/RSKA}]\ [\mathrm{SosA}] + k_{6b}\ [\mathrm{C_{P90/RSK-Sos}}] - k_{10f}\ [\mathrm{SosA}]\ [\mathrm{RasI}] + k_{10b}\ [\mathrm{C_{Sos-Ras}}] + k_{11f}\ [\mathrm{C_{Sos-Ras}}] \tag{18}$$

$$\frac{d}{dt}[\mathrm{C_{Erk-P90/RSK}}] = k_{8f}\ [\mathrm{ErkA}]\ [\mathrm{P90/RSKI}] - k_{8b}\ [\mathrm{C_{Erk-P90/RSK}}] - k_{9f}\ [\mathrm{C_{Erk-P90/RSK}}] \tag{19}$$

$$\frac{d}{dt}[\mathrm{C_{Ras-Raf1}}] = k_{14f}\ [\mathrm{RasA}]\ [\mathrm{Raf1I}] - k_{14b}\ [\mathrm{C_{Ras-Raf1}}] - k_{15f}\ [\mathrm{C_{Ras-Raf1}}] \tag{20}$$

$$\frac{d}{dt}[\mathrm{bEGFR}] = k_{0f}\ [\mathrm{EGF}][\mathrm{fEGFR}] - k_{0b}\ [\mathrm{bEGFR}] - k_{2f}\ [\mathrm{bEGFR}]\ [\mathrm{SosI}] + k_{2b}\ [\mathrm{C_{bEGFR-Sos}}] + k_{3f}\ [\mathrm{C_{bEGFR-Sos}}] - k_{34f}\ [\mathrm{bEGFR}]\ [\mathrm{PI3KI}] + k_{34b}\ [\mathrm{C_{bEGFR-PI3K}}] + k_{35f}\ [\mathrm{C_{bEGFR-PI3K}}] \tag{21}$$

$$\frac{d}{dt}[\text{Raf1A}] = k_{15f}\ [\text{C}_{\text{Ras-Raf1}}] - k_{16f}\ [\text{Raf1PPtase}]\ [\text{Raf1A}] + k_{16b}\ [\text{C}_{\text{Raf1PPtase-Raf1}}] - k_{18f}\ [\text{Akt/PKBA}]\ [\text{Raf1A}] + k_{18b}\ [\text{C}_{\text{Akt-Raf1}}] - k_{24f}\ [\text{Raf1A}]\ [\text{MekI}] + k_{24b}\ [\text{C}_{\text{Raf1-Mek}}] + k_{25f}\ [\text{C}_{\text{Raf1-Mek}}] \quad (22)$$

$$\frac{d}{dt}[\text{C}_{\text{Mek-Erk}}] = k_{30f}\ [\text{MekA}]\ [\text{ErkI}] - k_{30b}\ [\text{C}_{\text{Mek-Erk}}] - k_{31f}\ [\text{C}_{\text{Mek-Erk}}] \quad (23)$$

$$\frac{d}{dt}[\text{C}_{\text{RapGap-Rap1}}] = k_{44f}\ [\text{RapGapA}]\ [\text{Rap1A}] - k_{44b}\ [\text{C}_{\text{RapGap-Rap1}}] - k_{45f}\ [\text{C}_{\text{RapGap-Rap1}}] \quad (24)$$

$$\frac{d}{dt}[\text{SosI}] = -k_{2f}\ [\text{bEGFR}]\ [\text{SosI}] + k_{2b}\ [\text{C}_{\text{bEGFR-Sos}}] - k_{4f}\ [\text{bNGFR}]\ [\text{SosI}] + k_{4b}\ [\text{C}_{\text{bNGFR-Sos}}] + k_{7f}\ [\text{C}_{\text{P90/RSK-Sos}}] \quad (25)$$

$$\frac{d}{dt}[\text{RapGapA}] = -k_{44f}\ [\text{RapGapA}]\ [\text{Rap1A}] + k_{44b}\ [\text{C}_{\text{RapGap-Rap1}}] + k_{45f}\ [\text{C}_{\text{RapGap-Rap1}}] \quad (26)$$

$$\frac{d}{dt}[\text{C}_{\text{Akt-Raf1}}] = k_{18f}\ [\text{Akt/PKBA}]\ [\text{Raf1A}] - k_{18b}\ [\text{C}_{\text{Akt-Raf1}}] - k_{19f}\ [\text{C}_{\text{Akt-Raf1}}] \quad (27)$$

$$\frac{d}{dt}[\text{fEGFR}] = -k_{0f}\ [\text{EGF}]\ [\text{fEGFR}] + k_{0b}\ [\text{bEGFR}] \quad (28)$$

$$\frac{d}{dt}[\text{Raf1PPtase}] = -k_{16f}\ [\text{Raf1PPtase}]\ [\text{Raf1A}] + k_{16b}\ [\text{C}_{\text{Raf1PPtase-Raf1}}] + k_{17f}\ [\text{C}_{\text{Raf1PPtase-Raf1}}] - k_{22f}\ [\text{Raf1PPtase}]\ [\text{BRafA}] + k_{22b}\ [\text{C}_{\text{Raf1PPtase-BRaf}}] + k_{23f}\ [\text{C}_{\text{Raf1PPtase-BRaf}}] \quad (29)$$

$$\frac{d}{dt}[\text{C}_{\text{C3G-Rap1}}] = k_{42f}\ [\text{C3GA}]\ [\text{Rap1I}] - k_{42b}\ [\text{C}_{\text{C3G-Rap1}}] - k_{43f}\ [\text{C}_{\text{C3G-Rap1}}] \quad (30)$$

$$\frac{d}{dt}[\text{Rap1A}] = -k_{20f}\ [\text{Rap1A}]\ [\text{BRafI}] + k_{20b}\ [\text{C}_{\text{Rap1}_\text{B}\text{Raf}}] + k_{21f}\ [\text{C}_{\text{Rap1}_\text{B}\text{Raf}}] + k_{43f}\ [\text{C}_{\text{C3G-Rap1}}] - k_{44f}\ [\text{RapGapA}]\ [\text{Rap1A}] + k_{44b}\ [\text{C}_{\text{RapGap-Rap1}}] \quad (31)$$

$$\frac{d}{dt}[\text{MekI}] = -k_{24f}\ [\text{Raf1A}]\ [\text{MekI}] + k_{24b}\ [\text{C}_{\text{Raf1-Mek}}] - k_{26f}\ [\text{BRafA}]\ [\text{MekI}] + k_{26b}\ [\text{C}_{\text{BRaf-Mek}}] + k_{29f}\ [\text{C}_{\text{PP2A-Mek}}] \quad (32)$$

$$\frac{d}{dt}[\text{C}_{\text{Raf1-Mek}}] = k_{24f}\ [\text{Raf1A}]\ [\text{MekI}] - k_{24b}\ [\text{C}_{\text{Raf1-Mek}}] - k_{25f}\ [\text{C}_{\text{Raf1-Mek}}] \quad (33)$$

$$\frac{d}{dt}[\text{C}_{\text{Sos-Ras}}] = k_{10f}\ [\text{SosA}]\ [\text{RasI}] - k_{10b}\ [\text{C}_{\text{Sos-Ras}}] - k_{11f}\ [\text{C}_{\text{Sos-Ras}}] \quad (34)$$

$$\frac{d}{dt}[\text{P90/RSKA}] = -k_{6f}\ [\text{P90/RSKA}]\ [\text{SosA}] + k_{6b}\ [\text{C}_{\text{P90/RSK-Sos}}] + k_{7f}\ [\text{C}_{\text{P90/RSK-Sos}}] + k_{09f}\ [\text{C}_{\text{Erk-P90/RSK}}] \quad (35)$$

$$\frac{d}{dt}[\text{C}_{\text{PP2A-Erk}}] = k_{32f}\ [\text{PP2AA}]\ [\text{ErkA}] - k_{32b}\ [\text{C}_{\text{PP2A-Erk}}] - k_{33f}\ [\text{C}_{\text{PP2A-Erk}}] \quad (36)$$

$$\frac{d}{dt}[\text{C}_{\text{bEGFR-PI3K}}] = k_{34f}\ [\text{bEGFR}]\ [\text{PI3KI}] - k_{34b}\ [\text{C}_{\text{bEGFR-PI3K}}] - k_{35f}\ [\text{C}_{\text{bEGFR-PI3K}}] \quad (37)$$

$$\frac{d}{dt}[\text{PI3KA}] = k_{35f}\ [\text{C}_{\text{bEGFR-PI3K}}] + k_{37f}\ [\text{C}_{\text{Ras-PI3K}}] - k_{38f}\ [\text{PI3KA}]\ [\text{Akt/PKBI}] + k_{38b}\ [\text{C}_{\text{PI3K-Akt}}] + k_{39f}\ [\text{C}_{\text{PI3K-Akt}}] \quad (38)$$

$$\frac{d}{dt}[\text{RasGapA}] = -k_{12f}\ [\text{RasGapA}]\ [\text{RasA}] + k_{12b}\ [\text{C}_{\text{RasGap-Ras}}] + k_{13f}\ [\text{C}_{\text{RasGap-Ras}}] \quad (39)$$

$$\frac{d}{dt}[\text{BRafA}] = k_{21f}\ [\text{C}_{\text{Rap1}_\text{B}\text{Raf}}] - k_{22f}\ [\text{Raf1PPtase}]\ [\text{BRafA}] + k_{22b}\ [\text{C}_{\text{Raf1PPtase-BRaf}}] - k_{26f}\ [\text{BRafA}]\ [\text{MekI}] + k_{26b}\ [\text{C}_{\text{BRaf-Mek}}] + k_{27f}\ [\text{C}_{\text{BRaf-Mek}}] \quad (40)$$

$$\frac{d}{dt}[\text{C}_{\text{bEGFR-Sos}}] = k_{2f}\ [\text{bEGFR}]\ [\text{SosI}] - k_{2b}\ [\text{C}_{\text{bEGFR-Sos}}] - k_{3f}\ [\text{C}_{\text{bEGFR-Sos}}] \quad (41)$$

$$\frac{d}{dt}[\text{Rap1I}] = -k_{42f}\ [\text{C3GA}]\ [\text{Rap1I}] + k_{42b}\ [\text{C}_{\text{C3G-Rap1}}] + k_{45f}\ [\text{C}_{\text{RapGap-Rap1}}] \quad (42)$$

$$\frac{d}{dt}[\text{ErkA}] = -k_{8f}\ [\text{ErkA}]\ [\text{P90/RSKI}] + k_{8b}\ [\text{C}_{\text{Erk-P90/RSK}}] + k_{9f}\ [\text{C}_{\text{Erk-P90/RSK}}] \quad (43)$$

$$+ k_{31f}\ [\mathrm{C_{Mek-Erk}}] - k_{32f}\ [\mathrm{PP2AA}]\ [\mathrm{ErkA}] + k_{32b}\ [\mathrm{C_{PP2A-Erk}}]$$

$$\frac{d}{dt}[\mathrm{fNGFR}] = -k_{1f}\ [\mathrm{NGF}]\ [\mathrm{fNGFR}] + k_{1b}\ [\mathrm{bNGFR}] \tag{44}$$

$$\frac{d}{dt}[\mathrm{C3GI}] = -k_{40f}\ [\mathrm{bNGFR}]\ [\mathrm{C3GI}] + k_{40b}\ [\mathrm{C_{bNGFR-C3G}}] \tag{45}$$

$$\frac{d}{dt}[\mathrm{Raf1I}] = -k_{14f}\ [\mathrm{RasA}]\ [\mathrm{Raf1I}] + k_{14b}\ [\mathrm{C_{Ras-Raf1}}] + k_{17f}\ [\mathrm{C_{Raf1PPtase-Raf1}}] + k_{19f}\ [\mathrm{C_{Akt-Raf1}}] \tag{46}$$

$$\frac{d}{dt}[\mathrm{PI3KI}] = -k_{34f}\ [\mathrm{bEGFR}]\ [\mathrm{PI3KI}] + k_{34b}\ [\mathrm{C_{bEGFR-PI3K}}] - k_{36f}\ [\mathrm{RasA}]\ [\mathrm{PI3KI}] + k_{36b}\ [\mathrm{C_{Ras-PI3K}}] \tag{47}$$

$$\frac{d}{dt}[\mathrm{BRafI}] = -k_{20f}\ [\mathrm{Rap1A}]\ [\mathrm{BRafI}] + k_{20b}\ [\mathrm{C_{Rap1_BRaf}}] + k_{23f}\ [\mathrm{C_{Raf1PPtase-BRaf}}] \tag{48}$$

$$\frac{d}{dt}[\mathrm{C_{Rap1_BRaf}}] = k_{20f}\ [\mathrm{Rap1A}]\ [\mathrm{BRafI}] - k_{20b}\ [\mathrm{C_{Rap1_BRaf}}] - k_{21f}\ [\mathrm{C_{Rap1_BRaf}}] \tag{49}$$

$$\frac{d}{dt}[\mathrm{RasA}] = k_{11f}\ [\mathrm{C_{Sos-Ras}}] - k_{12f}\ [\mathrm{RasGapA}]\ [\mathrm{RasA}] + k_{12b}\ [\mathrm{C_{RasGap-Ras}}] - k_{14f}\ [\mathrm{RasA}]\ [\mathrm{Raf1I}] + k_{14b}\ [\mathrm{C_{Ras-Raf1}}] + k_{15f}\ [\mathrm{C_{Ras-Raf1}}] - k_{36f}\ [\mathrm{RasA}]\ [\mathrm{PI3KI}] + k_{36b}\ [\mathrm{C_{Ras-PI3K}}] + k_{37f}\ [\mathrm{C_{Ras-PI3K}}] \tag{50}$$

$$\frac{d}{dt}[\mathrm{RasI}] = -k_{10f}\ [\mathrm{SosA}]\ [\mathrm{RasI}] + k_{10b}\ [\mathrm{C_{Sos-Ras}}] + k_{13f}\ [\mathrm{C_{RasGap-Ras}}] \tag{51}$$

$$\frac{d}{dt}[\mathrm{C_{PP2A-Mek}}] = k_{28f}\ [\mathrm{PP2AA}]\ [\mathrm{MekA}] - k_{28b}\ [\mathrm{C_{PP2A-Mek}}] - k_{29f}\ [\mathrm{C_{PP2A-Mek}}] \tag{52}$$

$$\frac{d}{dt}[\mathrm{C_{PI3K-Akt}}] = k_{38f}\ [\mathrm{PI3KA}]\ [\mathrm{Akt/PKBI}] - k_{38b}\ [\mathrm{C_{PI3K-Akt}}] - k_{39f}\ [\mathrm{C_{PI3K-Akt}}] \tag{53}$$

$$\frac{d}{dt}[\mathrm{C3GA}] = k_{41f}\ [\mathrm{C_{bNGFR-C3G}}] - k_{42f}\ [\mathrm{C3GA}]\ [\mathrm{Rap1I}] + k_{42b}\ [\mathrm{C_{C3G-Rap1}}] + k_{43f}\ [\mathrm{C_{C3G-Rap1}}] \tag{54}$$
